# From Paddock to Foal: Prevalence and Genotypic Diversity of *Rhodococcus equi* on Stud Farms in Türkiye

**DOI:** 10.3390/vetsci13010072

**Published:** 2026-01-10

**Authors:** Zeynep Yerlikaya, Burcu Karagülle, Barış Otlu, Adile Muz

**Affiliations:** 1 Department of Microbiology, Faculty of Veterinary Medicine, Firat University, Elazig 23200, Türkiye; 2 Department of Microbiology, Faculty of Medicine, İnönü University, Malatya 44280, Türkiye

**Keywords:** *Rhodococcus equi*, *vapA*, PFGE, PCR, antimicrobial resistance, foal pneumonia

## Abstract

Pneumonia caused by the facultative intracellular pathogen *Rhodococcus equi* stands out as one of the most significant infections associated with a notably high mortality rate in foals worldwide. Limited therapeutic options and inadequate control and prevention measures result in substantial economic losses, underscoring the need for enhanced interventions. A cross-sectional, multi-province study was conducted on racehorse-breeding farms in Türkiye to estimate prevalence and index virulence, assess relatedness, and summarize antimicrobial susceptibility within a farm management context. Nasal and fecal swabs and environmental samples (soil and water) were cultured and confirmed; virulence was assessed with *vapA*-specific PCR, genetic relationship determined with PFGE, and antimicrobial susceptibility using disk diffusion. *R. equi* was detected in 10% of nasal swabs, 22.9% of fecal swabs, 29.4% of soil samples, and 5.9% of water samples; 46.2% of confirmed isolates were *vapA*-positive. Susceptibility patterns were favorable overall, with frequent ampicillin resistance, infrequent resistance to macrolides and rifampin, rare multidrug resistance, and no vancomycin resistance was detected. PFGE demonstrated substantial genotypic diversity, with 12 clusters and 29 distinct pulsotypes. Farm-level observations were exploratory. More frequent mechanical paddock cleaning coincided with the absence of foal deaths, and *vapA*-positive isolates were observed on farms with prior infection. Taken together, these findings support routine paddock hygiene, prompt isolation of clinically affected foals, culture-guided therapy, and continued surveillance, and they indicate a need for longitudinal and genomic studies to evaluate the impact.

## Simple Summary

*Rhodococcus equi* can cause severe pneumonia in young foals, putting their health and survival at risk and challenging the sustainability of horse-breeding farms. During the foaling season, 20 stud farms in five provinces of Türkiye were visited to learn how often this bacterium was present, how it was distributed across animals and the environment, and which routine farm practices might matter. Nasal and fecal swabs from foals and samples from soil and water were collected. *R. equi* was detected more often in fecal swabs than in nasal swabs, was common in soil, and was rare in water. About half of the bacteria we found carried features linked to disease in foals. Most isolates remained susceptible to commonly used antimicrobials, and multidrug resistance was uncommon. Farms reporting more frequent paddock cleaning and separation of clinically affected foals tended to report fewer deaths. Together with culture-guided therapy for affected foals and ongoing surveillance, these practical measures may help reduce disease impact and protect foal health.

## 1. Introduction

*Rhodococcus equi* (syn. *Prescottella equi*), first isolated from foals with suppurative bronchopneumonia by Magnusson in 1923, is a Gram-positive actinobacterium that behaves as a facultative intracellular pathogen of macrophages and is commonly associated with paddock soil, dust, and equine feces on breeding farms [1]. Taxonomically, *R. equi* is placed in the family Nocardiaceae within the order Mycobacteriales; current nomenclatural resources list *Prescottella equi* as a homotypic synonym of *R.equi*, and recent field consensus supports continued use of *Rhodococcus equi* in veterinary contexts [2,3,4,5].

*R. equi* is distributed on stud farms globally, in America, Europe, Asia, and Australia [6]. Farm-level epidemiology ranges from endemic to sporadic patterns, with marked variation in environmental detection and foal positivity between premises and regions. Environmental contamination is commonly demonstrated in soils and paddock dust, and shedding in feces by foals and adults contributes to on-farm contamination and exposure pressure [7].

In foals two to six months old, *R. equi* is a leading cause of severe bronchopneumonia that can progress to pyogranulomatous lesions with regional lymphadenitis, resulting in morbidity, case fatality, reduced performance, and economic losses related to treatment costs, prolonged convalescence, and decreased sale value [8,9]. Opportunistic infections are also reported in other domestic species and in immunocompromised humans, although the principal veterinary burden remains in young foals [10,11].

Risk is multifactorial and reflects the interplay of environment, management, host, and pathogen. On-farm exposure is driven by the environmental load of *R. equi* in soil and dust, which is amplified under arid, dusty conditions and modulated by paddock hygiene, manure management, stocking density, and the movement of animals, staff, and equipment between premises. Host susceptibility varies with foal age and immune status, including the adequacy of colostral transfer, and farms with a prior history of *R. equi* disease often show ongoing environmental contamination with recurrent clinical cases [12,13,14]. At the pathogen level, virulence depends on a cell surface protein encoded on the virulence plasmid pathogenicity island, the 15–17 kDa virulence-associated protein A (VapA), which diverts phagosome maturation and creates a hospitable intracellular niche in macrophages [15]. Isolates carrying the equine-associated virulence plasmid (pVAPA) and its marker *vapA* are considered virulent in foals, and *vapA* detection is widely used to identify equine-type virulent isolates in epidemiological studies of environmental burden and farm-level disease patterns [16].

Definitive diagnosis in foals requires airway sampling. Culture and polymerase chain reaction (PCR) performed on transtracheal or tracheal aspirates provide etiologic confirmation. Thoracic ultrasonography is widely used on endemic farms to screen for and monitor subclinical pulmonary lesions [17]. The recommended regimen is a macrolide with rifampin, typically for weeks; such prolonged therapy is associated with adverse effects and with selection pressure for macrolide/rifampin non-susceptibility, underscoring the need to pair treatment with management strategies that reduce exposure and transmission [18]. There is currently no licensed vaccine in routine use, and evidence for prophylactic hyperimmune plasma is mixed [19].

Environmental control measures are variably implemented and difficult to standardize across farms. *R. equi* is widely present in soil and in the manure of herbivores, which makes control and prevention intrinsically challenging [20]. Experimental and field observations indicate that *R. equi* can persist in the environment for prolonged periods, with survival in soil reported for up to approximately 24 months [21]. In this context, complete eradication is not a realistic near-term goal; emphasis should be placed on measures that reduce exposure and transmission risk at the farm level [17,19].

Because environmental persistence complicates on-farm control, strain-level typing helps distinguish local persistence from repeated introductions and informs transmission links. Multiple VapA plasmid types have been described across North America, Europe, Australia, Africa, Korea, Japan, and Türkiye, classified with RFLP as 85 kb types I-IV, 87 kb types I-III, and 90 kb types I-V [22,23]. While RFLP defines plasmid types, PFGE (Pulsed Field Gel Electrophoresis) has been widely used to infer strain relatedness at farm and regional scales, and this approach is increasingly complemented by whole genome sequencing for higher resolution [24,25]. Comparative analyses of virulent and avirulent *R. equi* isolates have helped resolve genotypic lineages across hosts and geographic regions [26,27].

Against this background, this cross-sectional multi-province field study was designed on stud farms in Türkiye to generate baseline data on the burden and molecular epidemiology of *R. equi* and to examine farm management practices in relation to microbiological outcomes. Clinical and environmental specimens were obtained and analyzed; virulence was inferred from *vapA* detection; clonal relatedness among virulent isolates was evaluated; antimicrobial susceptibility was profiled; and management and hygiene practices were documented using a structured survey and examined for associations with positivity and virulence indicators. These data aim to inform practical control measures and provide a regional reference for molecular epidemiology of *R. equi*.

## 2. Materials and Methods

### 2.1. Study Area, Design, and Sampling

**Figure 1:**
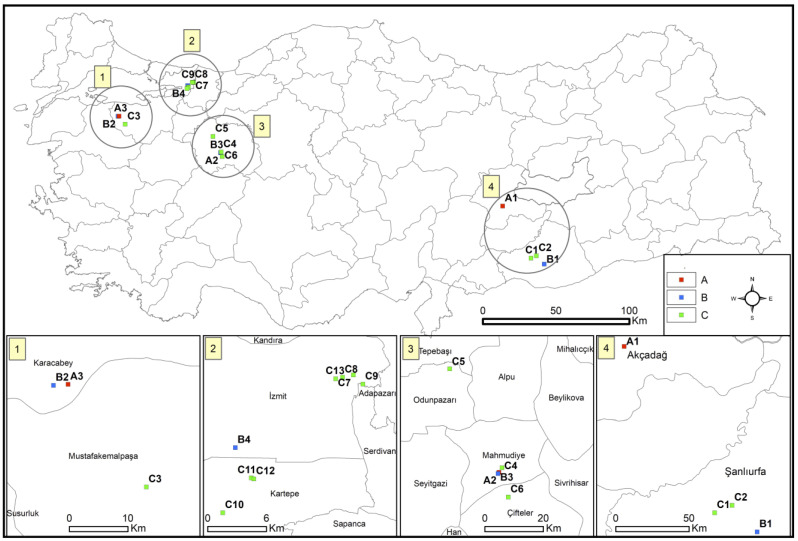
Sampling locations of 20 stud farms across five provinces in Türkiye (Malatya, Bursa, İzmit, Eskişehir, and Şanlıurfa). Each marker denotes a stud farm; codes beginning with A or B indicate farms affiliated with institutional enterprises (*n* = 7), whereas codes beginning with C denote private farms (*n* = 13). The national panel shows provincial context; numbered inset maps provide enlarged regional views with scale bars (km) and a north arrow.

This cross-sectional study was conducted during the foaling season from 1 April to 1 June 2017. The study area comprised the five horse-breeding provinces of Malatya, Bursa, İzmit, Eskişehir, and Şanlıurfa in Türkiye. Nasal and fecal swabs were collected from foals aged 2–6 months, and soil and water were sampled from paddocks at participating stud farms. In total, 20 stud farms were included (7 institutional and 13 private; Figure 1). A brief structured questionnaire was administered to farm veterinarians to document farm management and facility characteristics (Table S1). At three institutional farms (coded A), environmental sampling (soil and water) was not permitted; only nasal and fecal swabs were collected.

Farms were enrolled based on willingness to participate and logistical feasibility during the foaling season; no stratified selection by farm size was applied. Within farms, sampling targeted eligible foals present at the time of the visit, so the number sampled per farm varied by access and foal availability.

Standardized farm census data (total horses present during the foaling season), soil classification, and meteorological variables (e.g., rainfall) were not systematically recorded as part of the study design.

The required sample size was estimated in Epi Info for a cross-sectional survey assuming an expected prevalence of 20%, a 95% confidence level, and an absolute precision of 5% (margin of error), yielding a minimum of 246 animals; 428 foals were ultimately sampled. The study population was classified into three groups: (i) healthy-appearing foals, (ii) clinically suspect foals, and (iii) dams of suspect foals. The number of animals sampled per farm is reported in the Supplementary Materials (Table S2).

Foals were gently restrained for sampling. Sterile charcoal Amies transport swabs (LP Italiana, Milan, Italy) were used for both nasal and fecal sampling. For nasal sampling, the swab was advanced into the nostril as far as practicable without causing discomfort and was immediately returned to the transport tube. Fecal swabs were obtained by inserting a sterile swab per rectum and returning it to the transport tube.

Where permitted, soil and water were sampled from paddocks accessed by foals. For each farm, soil was collected as one composite sample by pooling multiple subsamples obtained from several paddock micro-locations with high animal contact (e.g., around feeding and watering points, shelter or standing areas, and manure-rich or heavily used sections) into a sterile container. Water was collected aseptically into a sterile container from the primary drinking-water source available to foals and processed as one water sample per farm.

All specimens were transported to the laboratory under cold-chain conditions. Due to the high number of specimens received during each sampling round, samples were snap-frozen in liquid nitrogen upon arrival and subsequently stored at −80 °C until bacteriological processing. Before culture, samples were thawed on ice and processed immediately; freeze/thaw was limited to a single cycle.

### 2.2. Bacterial Isolation

Samples were streaked onto NANAT selective agar (nalidixic acid–novobiocin–cycloheximide–potassium tellurite). Soil samples were processed as described by Takai et al. (1991) [28]. Plates were incubated aerobically at 37 °C for 48–72 h. Colonies exhibiting a gray, shiny, mucoid morphology were subcultured onto blood agar for phenotypic assessment and retained for molecular identification. The ATCC 33701 strain was used as a positive control for optimization of growth on the selective medium.

### 2.3. Molecular Identification

Genomic DNA was extracted using a classical phenol-chloroform method. Species identity was initially confirmed with PCR targeting the *Rhodococcus equi* 16S rRNA gene, yielding a 450 bp amplicon, using primers 5′-GGTCTAATACCGGATATGAGCTCCTGTC-3′ (forward) and 5′-CGCAAGCTTGGGGTTGAGCCCCAA-3′ (reverse) [29]. Reactions (final volume 25 μL) contained 200 μM each dNTP, 2.5 μL 10× PCR buffer, 1.5 mM MgCl_2_, 0.6 U Taq DNA polymerase, 10 pmol of each primer, and 2 μL template DNA. Cycling conditions were 95 °C for 5 min; 35 cycles of 94 °C for 1 min, 52 °C for 1 min, and 72 °C for 1 min; followed by 72 °C for 10 min and a hold at 4 °C.

Detection of the virulence gene *vapA* employed primers 5′-GACTCTTCACAAGACGGT-3′ (forward) and 5′-TAGGCGTTGTGCCAGCTA-3′ (reverse) [30], yielding a 563 bp product. Reaction components were the same as above. Cycling conditions were 94 °C for 2 min; 40 cycles of 94 °C for 1.5 min, 57 °C for 1 min, and 72 °C for 2 min; final extension 72 °C for 10 min; hold at 4 °C. Amplicons were resolved on 2% agarose gels and visualized with UV transillumination. *R. equi* ATCC 33701 was used as a positive control.

### 2.4. Antimicrobial Susceptibility Testing

Antimicrobial susceptibility testing was determined using the disk diffusion method in accordance with Clinical & Laboratory Standards Institute (CLSI) guidelines [31]. Briefly, bacterial suspensions were adjusted to a 0.5 McFarland standard and evenly swabbed onto Mueller–Hinton agar supplemented with 5% defibrinated sheep blood. Oxoid disks (Basingstoke, UK) containing ampicillin (10 µg), clarithromycin (15 µg), erythromycin (15 µg), gentamicin (30 µg), rifampin (30 µg), streptomycin (25 µg), tetracycline (30 µg), and vancomycin (30 µg) were applied, and plates were incubated aerobically at 37 °C for 24–48 h. Zone diameters were interpreted and categorized as susceptible, intermediate, or resistant. In the absence of *Rhodococcus equi*-specific breakpoints, interpretive criteria for *Staphylococcus* spp. were used as surrogate thresholds [32].

### 2.5. Pulse Field Gel Electrophoresis (PFGE)

PFGE was performed as described by Morton et al. [33] and Witkowski et al. [34]. Briefly, *vapA*-positive *R. equi* colonies were inoculated into 5 mL Brain Heart Infusion (BHI) broth and incubated at 37 °C with shaking at 225 rpm until the culture reached an OD_600_ of 0.95. Cells from 1 mL of culture were harvested (16,250× *g*, 10 min), washed three times in 1 M NaCl-10 mM Tris-HCl (pH 7.5), and resuspended in 100 µL of the same buffer. Suspensions were mixed 1:1 with molten 1.2% (*w*/*v*) low-melting agarose (SeaKem Gold LMP, Lonza, Rockland, ME, USA) and dispensed into 100 µL plug molds. Plugs were incubated in lysis buffer (100 mM Tris-HCl, 10 mM EDTA [pH 8.0], 0.5 M NaCl, 20% [*w*/*v*] sucrose, 5 mg mL^−1^ lysozyme) for 24 h at 37 °C. Then, 110 µL of 10% sodium N-lauroyl sarcosinate was added, and incubation continued for a further 24 h at 37 °C. The lysis buffer was replaced with ESP buffer (0.5 M EDTA [pH 9.2], 1% N-lauroyl sarcosinate, 1 mg mL^−1^ proteinase K), and the plugs were incubated at 50 °C for 50–54 h. Genomic DNA embedded in the plugs was digested with *AseI* (15–20 U per plug; Fermentas, Vilnius, Lithuania) at 37 °C for 16 h. Macrorestriction fragments were resolved on a CHEF-DR II pulsed-field system (Bio-Rad Laboratories, Hercules, California, USA) in 1.5% agarose at 14 °C, 200 V, with an included angle of 120° for a total of 22 h. Two linear switch-time ramps were used: 6–15 s for 7 h, followed by 23–40 s for 15 h. Digital gel images were analyzed with GelCompar II (v3.0; Applied Maths, Sint-Martens-Latem, Belgium). Similarity was calculated using the Dice coefficient, and clustering was performed using the UPGMA (Unweighted Pair Group Method with Arithmetic Mean); band-matching parameters were set to 1.0% position tolerance and 1.0% optimization.

### 2.6. Statistical Analysis

All analyses were conducted in IBM SPSS Statistics version 22.0 (IBM Corp., Armonk, NY, USA). Differences in proportions were evaluated using Pearson’s chi-square (*χ*^2^) test; Fisher’s exact test was applied where appropriate. Correlations between farm-level variables were assessed with Spearman’s rank correlation (ρ). All tests were two-sided, and *p* < 0.05 was considered statistically significant.

## 3. Results

### 3.1. Recovery, Distribution, and Virulence Gene Carriage of R. equi

**Figure 2:**
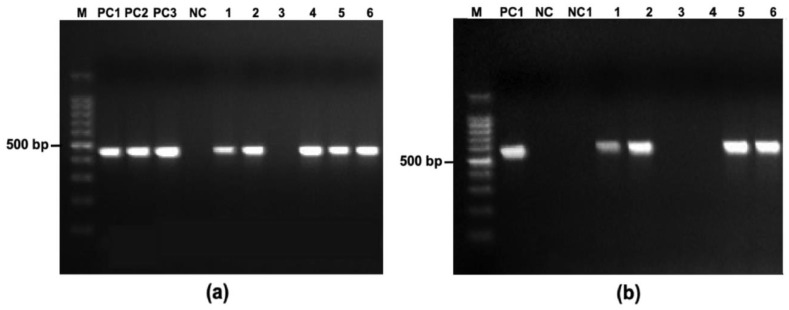
(**a**) Species confirmation by 16S rRNA PCR (450 bp) on an ethidium-bromide-stained agarose gel; lanes: M, 100 bp DNA ladder (Invitrogen); PC1, *R. equi* ATCC 6939; PC2, *R. equi* ATCC 33701; PC3, *R. equi* 103S+; NC, no-template control; 1-6, DNA from suspected colonies. (**b**) Detection of the virulence gene *vapA* (563 bp) in isolates previously confirmed as *R. equi*; lanes: M, 100 bp ladder; PC1, *R. equi* ATCC 33701 (*vapA+*); NC, *R. equi* ATCC 6939 (*vapA−*); NC1, no-template control; 1-6, field isolates confirmed as *R.equi*.

Across 890 specimens plated on NANAT selective agar, 150 colonies with a gray, shiny, mucoid morphology were recovered and subcultured. Following DNA extraction, *R. equi*-specific 16S rRNA PCR confirmed 147 isolates as *R. equi* and 46.3% (68/147) of confirmed isolates carried the *vapA* gene (Figure 2).

**Figure 3:**
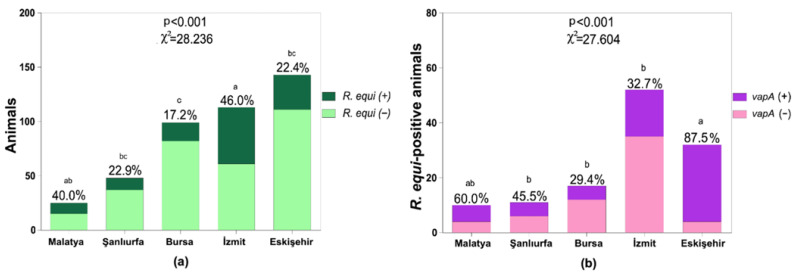
Provincial distribution of *R. equi* positivity and *vapA* carriage among sampled horses. (**a**) *R. equi* positivity by province. Horses were classified as *R. equi*-positive if *R. equi* was detected in at least one of the paired nasal and/or fecal swabs. Bars show percentages; different letters (a,b,c) indicate statistically significant differences between provinces, whereas bars sharing a letter do not differ significantly (Pearson *χ*^2^; pairwise Fisher’s exact tests, α = 0.05). (**b**) Percentage of *R. equi*-positive horses with *vapA*-positive isolates by province. Bars show percentages among *R. equi*-positive animals; different letters (a,b) indicate statistically significant differences (Pearson *χ*^2^; pairwise Fisher’s exact tests, α = 0.05).

At the sample level, *R. equi* was detected in 10.0% (43/428) of nasal swabs and 22.9% (98/428) of fecal swabs (*χ*^2^ test, *p* < 0.001). Environmental sampling yielded positive detection from 29.4% (5/17) of soil samples and 5.9% (1/17) of water samples. By province, the proportion of positive samples among those collected was highest in İzmit, at 26.4% (64/242), and lowest in Bursa, at 9.4% (19/202) (*p* < 0.001; Table 1). At the animal level, positivity in either nasal or fecal swab was identified in 28.5% (122/428) of horses, ranging from 46.0% (52/113) in İzmit to 17.2% (17/99) in Bursa (*p* < 0.001; Figure 3a). In a subset of 36 clinically suspect foals and their dams, concomitant positivity was observed for 7 pairs; there was no significant difference between foals and dams (*p* = 0.182; Table S3). These between-province differences are presented descriptively and should be interpreted cautiously because sampling intensity was not uniform across provinces. Variation in the number of participating farms and sampled animals captures underlying farm-level heterogeneity and field access constraints across provinces.

Among *vapA*-positive isolates (n = 68), the source distribution was 27.9% (19/68) nasal, 69.1% (47/68) fecal, and 2.9% (2/68) soil. The province-level prevalence of *vapA* among isolates was highest in Eskişehir (83.8%, 31/37) and lowest in İzmit (28.6%, 18/63), with significant differences across provinces (*χ*^2^ test, *p* < 0.001; Table 2). At the individual horse level, 50.0% (61/122) of *R. equi*-positive animals carried a *vapA*-positive (virulent) strain; provincial rates ranged from 87.5% (28/32) in Eskişehir to 29.4% (5/17) in Bursa (*p* < 0.001; Figure 3b). Provincial comparisons of *vapA* positivity are presented descriptively, as enrollment and sampling intensity differed between provinces and therefore limit province-level inference. The number of animals sampled per farm is summarized in Table S2.

### 3.2. Antimicrobial Susceptibility

According to disk diffusion, 43.5% of isolates were susceptible to all tested antibiotics. Non-susceptibility to at least one antibiotic was 45.6%, driven predominantly by single-drug resistance (39.4%), with smaller proportions showing dual ampicillin/streptomycin resistance (3.4%) or multidrug resistance (2.7%, defined as non-susceptibility to ≥3 classes) (Table 3). A further 10.8% exhibited intermediate-only profiles. Resistance was highest to ampicillin (45.5%), lower to streptomycin (7.6%), and infrequent for rifampin (3.4%), erythromycin (2.7%), clarithromycin (2.7%), and gentamicin (0.7%), while no resistance to vancomycin was detected (Table 4). Intermediate responses were infrequent (10.8%), occurring chiefly with ampicillin (*n* = 7) and rifampin (*n* = 3); sporadic intermediates were also observed for gentamicin and vancomycin, and in a few instances involved two agents in the same isolate.

To evaluate whether antimicrobial susceptibility differed according to virulence status, *vapA*-positive and *vapA*-negative isolates were compared using Fisher’s exact test, with results classified as susceptible or intermediate/resistant. No significant differences were observed between the two groups for rifampin (*p* = 0.341), erythromycin (*p* = 0.6864), clarithromycin (*p* = 0.1241), or ampicillin (*p* = 0.3229). Comparisons for the remaining antimicrobial agents were not prioritized because non-susceptibility was infrequent and resistant isolates were absent for some agents, limiting meaningful group-wise evaluation.

### 3.3. PFGE

**Figure 4:**
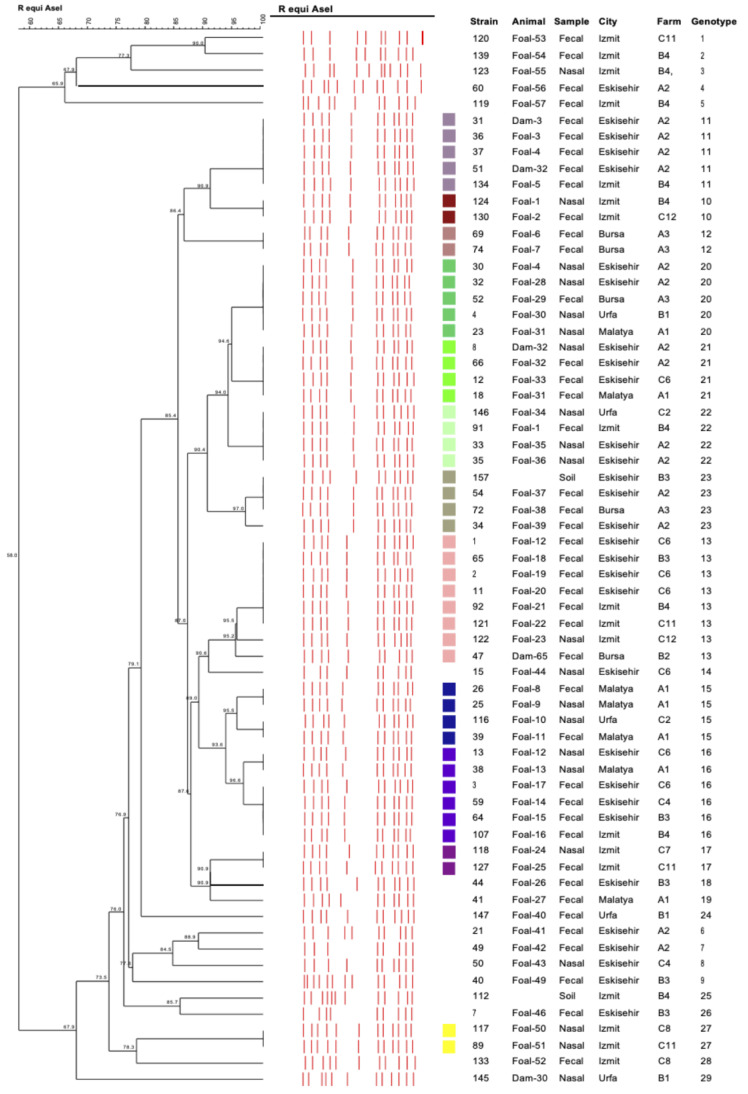
PFGE genotyping of *Rhodococcus equi* isolates from stud farms in Türkiye. Chromosomal DNA was digested with *AseI* and separated by PFGE. The dendrogram was generated in GelCompar II using the Dice coefficient and UPGMA clustering (position tolerance 1.0%, optimization 1.0%); the scale indicates percent similarity. Right-hand columns list isolate ID, host (foal/dam), specimen (nasal/fecal), province, and farm code; the colored strip denotes the pulsotype (genotype number). Clusters were defined at ≥90% similarity.

PFGE was performed to assess clonal relatedness among 65 *vapA*-positive *Rhodococcus* equi isolates collected from multiple provinces and farms. Digital images were analyzed in GelCompar II using the Dice similarity coefficient and UPGMA clustering, with band-matching parameters set to a position tolerance of 1.0% and optimization of 1.0%; clusters were defined at ≥90% similarity. The macrorestriction profiles resolved 29 pulsotypes and, at this threshold, yielded 12 clusters, while 17 isolates remained singletons; pairwise similarities spanned 58-100%. In total, 48/65 (73.8%) isolates belonged to clusters, with cluster sizes ranging from 2 to 8 isolates. The largest cluster (cluster 13) comprised 8 isolates, followed by cluster 16 (*n* = 6), cluster 11 (*n* = 5), and cluster 20 (*n* = 5), whereas clusters 10, 12, 17, and 27 each contained 2 isolates (Figure 4).

Control lanes using *Rhodococcus equi* ATCC 33701 were included for visualization and quality control and were excluded from clustering and similarity calculations. Isolates were run on multiple gels; therefore, inter-gel clustering was interpreted cautiously (Figure S1).

PFGE analysis demonstrated substantial diversity among *vapA*-positive isolates, with multiple clusters containing isolates from different farms and provinces (Figure 4). Several clusters showed patterns consistent with geographic proximity and enterprise-linked contact structures. Cluster 10 comprised two isolates (one nasal and one fecal) recovered from two different farms in İzmit located in close geographic proximity, consistent with local circulation of a shared pulsotype. Cluster 11 included five isolates, four of which originated from an Enterprise A farm in Eskişehir, while the remaining isolate originated from an Enterprise B farm in İzmit, indicating that related profiles could occur across different locations. Cluster 20 contained isolates from Enterprise A farms in Eskişehir, Bursa, and Malatya, alongside an isolate from an Enterprise B farm in Şanlıurfa, which aligns with the reported movement and contact networks within enterprise-affiliated premises. The largest group (cluster 13) included isolates from multiple farms across Eskişehir, İzmit, and Bursa, including both enterprise-affiliated and private (C) premises, supporting concurrent circulation of related pulsotypes across sites. In addition, Cluster 23 contained both fecal isolates and one soil isolate from an Enterprise B paddock in Eskişehir, indicating that similar pulsotypes could be recovered from both animal-derived and environmental specimens.

Within-animal comparisons further supported heterogeneity at the individual level: nasal and fecal isolates from the same foal were sometimes assigned to different PFGE clusters, whereas isolates from some clinically suspect foals and their dams clustered together (e.g., cluster 11). Taken together, these findings indicate concurrent circulation of multiple pulsotypes across farms and provinces, rather than dominance of a single farm-restricted clone (Figure 4).

### 3.4. Farm Management Characteristics and Exploratory Associations

A brief structured questionnaire on management and hygiene practices was administered to veterinarians at 17 stud farms. The questionnaire and coding schema are provided in Table S1. Aggregate frequencies and medians are summarized (Table 5), with farm-level responses reported in Table S4a,b. A prior history of *R. equi* on the premises was reported by 12/17 (70.6%) farms. Isolation of clinically affected foals during treatment was common (14/17, 82.4%), whereas routine hyperimmune plasma administration to all foals was uncommon (1/17, 5.9%). Bedding was typically wheat straw (16/17, 94.1%). Reported sanitation practices varied: Virkon S (4/17), lime powder (4/17), organic acid (1/17), none (4/17), and other products (4/17) (Table 5 and Table S4a). The median frequency of mechanical paddock cleaning was 12 times/year (range 0–24) and disinfection 1 time/year (0–5) (Table 5).

Outcomes and virulence signals are shown in Table S4b. Foal losses attributed to *R. equi* were reported by 10/17 (58.8%) farms. Virulent isolates were recovered from farms with a documented prior history of infection, and no virulent strains were detected on the five farms without such a history (Table S4b).

Exploratory correlations identified two notable associations (Table S5): a strong positive correlation between a prior farm history of *R. equi* (Q1) and reporting of foal deaths attributed to *R. equi* (Q10; ρ = 0.772, *p* < 0.01), and a positive correlation between more frequent mechanical paddock cleaning (Q14) and the absence of such deaths (Q10; ρ = 0.492, *p* < 0.05). These associations are descriptive and hypothesis-generating rather than causal.

## 4. Discussion

This study was designed to fill key gaps in the epidemiology of *Rhodococcus equi* on racehorse-breeding farms in Türkiye. Recovery across multiple specimen types was estimated, potential farm-level risk correlates were explored, and genetic relatedness among virulent isolates was assessed. By combining paired nasal and fecal swab culture with environmental sampling, molecular detection, PFGE typing of *vapA*-positive isolates, and antimicrobial susceptibility testing, pronounced farm-level heterogeneity and high genotypic diversity among virulent strains were observed. To our knowledge, PFGE-based genotyping data for virulent *R. equi* isolates from Türkiye are reported here for the first time, providing a molecular baseline for longitudinal surveillance and higher-resolution genomic studies aimed at clarifying transmission pathways and informing evidence-based farm management.

Against this background, the burden of *R. equi* was first characterized across the sampled specimen types to provide context for subsequent interpretation of virulence, genotyping, and susceptibility findings. *R. equi* was recovered from 10.0% of nasal swabs and 22.9% of fecal swabs, and from 29.4% of soil and 5.9% of water samples. This isolation profile is consistent with the ecology of *R. equi* on stud farms. The higher recovery from fecal than nasal swabs suggests that fecal shedding may be an important contributor to paddock contamination and environmental loading, while recovery from soil is consistent with the paddock environment and dust acting as key exposure reservoirs for susceptible foals [19]. In line with the view that soil and fecal contamination constitute the primary amplifying reservoirs, detection in water is often most plausibly interpreted as secondary or transient contamination (e.g., dust deposition or direct animal contact) rather than sustained persistence [35].

Where our nasal yield exceeded several prior reports [36,37], immediate placement of swabs into charcoal Amies transport medium, which is known to help preserve the viability of fastidious organisms during transport, may have contributed [38]. By contrast, comparatively lower yields in some matrices can plausibly be explained by differences in analytical sensitivity, particularly the absence of a pre-enrichment step and the use of swabs rather than bulk fecal material, both of which can limit the amount of organism captured and subsequently recovered by culture. More broadly, these observations are consistent with the methodological principle that recovery of *R. equi* from contaminated samples is shaped by the sampled matrix and by the selective/enrichment strategy applied, including the frequent use of selective media such as NANAT for environmental and fecal specimens [39,40]. Furthermore, since lower-airway sampling was not feasible under field conditions, nasal swab results are best interpreted as indicators of exposure rather than direct proxies for pneumonia prevalence.

Inter-province variation was noted in both overall *R. equi* recovery and *vapA* carriage; however, these differences are best interpreted as descriptive/ecological patterns rather than population-level contrasts. Farm participation and sampling intensity were inevitably uneven across provinces, and the number of foals available during the foaling season could not be fully anticipated in advance, resulting in imbalanced specimen numbers between locations. Furthermore, environmental sampling was not permitted on three institutional farms, limiting direct cross-province comparison of soil and water results. Province-level observations are therefore presented as hypothesis-generating signals to be evaluated in future studies with more standardized sampling density, fuller farm-level covariates, and longitudinal follow-up.

Foal virulence is largely determined by host-associated, plasmid-encoded factors, and the *vapA* gene (carried on equine-type pVAPA plasmids) is widely used as an epidemiologic marker of virulent *Rhodococcus equi* strains [15,16]. In the present study, 46.2% of confirmed isolates carried *vapA*, and virulent isolates were detected on 12 farms, while none were detected on 5 farms without a documented history of disease, in line with earlier observations that virulent strains tend to be recovered more often from endemic premises than from farms with sporadic or no recognized disease [22,35]. Importantly, detection of *vapA* in nasal or fecal swabs should be interpreted as evidence of exposure and/or colonization rather than as a site-specific indicator of pulmonary infection; diagnostic performance and clinical inference depend on specimen type and clinical context [41]. In this context, apparent differences in *vapA* carriage across provinces are presented descriptively and interpreted in light of farm-level heterogeneity and sampling constraints.

At the provincial level, *vapA* positivity appeared higher in Eskişehir and lower in İzmit despite frequent recovery of *R. equi* overall. Eskişehir farms were more closely spaced and included premises within an institutional network, which could plausibly increase connectivity through animal movements and shared personnel or equipment. In contrast, participating farms in İzmit were more geographically dispersed, a setting that may reduce opportunities for between-farm spread. These observations remain ecological hypotheses and should be evaluated in larger, longitudinal datasets that capture animal movement patterns and key farm- and environment-level variables.

To help interpret the marked farm-level heterogeneity observed in *R. equi* recovery and *vapA* carriage, management features were considered alongside other recognized predisposing domains. Rhodococcosis is widely regarded as multifactorial: susceptibility and exposure are shaped not only by hygiene practices but also by host and environmental determinants such as foal age and immune maturation, adequacy of passive transfer, stocking density and contact structure, airborne dust and microclimate, soil characteristics, animal movements and farm connectivity, and antimicrobial use patterns. These determinants have been linked to exposure intensity on endemic farms and to the ecology of virulent *R. equi* in air/airborne dust and soil [42,43]. Most of these determinants were not quantified in the present cross-sectional survey and therefore could not be assessed directly. 

Within the scope of the questionnaire, several farm practices with plausible relevance to exposure intensity and on-farm amplification were captured, including routine paddock manure removal/cleaning, disinfection frequency and product choice, isolation of clinically affected foals during treatment, and use of hyperimmune plasma. In exploratory analyses, more frequent mechanical paddock cleaning was associated with the absence of foal deaths attributed to *R. equi* (Spearman’s ρ = 0.492, *p* < 0.05; Table S5), which is compatible with the concept that reducing organic contamination may lower environmental loading. Conversely, failure to isolate clinically affected foals during treatment was reported at several facilities, a practice that could plausibly increase within-farm exposure for susceptible foals. A single farm that reported hyperimmune plasma administration to all foals and intensive hygiene practices did not yield *R. equi* during the sampling period. Evidence for hyperimmune plasma as a preventive tool remains mixed across studies, but its use on endemic farms is widely discussed and has been evaluated in controlled trials; therefore, the lack of culture recovery at a farm reporting hyperimmune plasma use and intensive hygiene is best treated as anecdotal and hypothesis-generating rather than as evidence of effect [44,45].

Additional questionnaire signals were compatible with farm- and animal-level influences that warrant more structured evaluation. For example, farms in which dams associated with affected/deceased foals had previously produced affected offspring raise the possibility of shared environmental exposure and maternal or genetic contributions, although pedigree-controlled longitudinal designs would be required to disentangle these mechanisms. Nutrition was not a prespecified endpoint, but contrasts in reported feeding strategies (e.g., a more protein-rich ration at one farm versus concentrate-only feeding at another) were noted and should be treated as hypothesis-generating until diet composition and passive transfer metrics (e.g., colostral and foal IgG) are measured prospectively [46].

Experimental work indicates that strong acidity reduces the survival of *R. equi* in vitro, supporting the principle that low pH conditions can limit persistence under laboratory conditions [47]. In our dataset, farms reporting use of Virkon S tended to have lower proportions of *vapA*-positive animals than farms reporting organic acid sanitation, whereas lime powder showed intermediate values. This pattern is consistent with equine biosecurity guidance that lists peroxygen disinfectants such as Virkon S among recommended options and emphasizes correct label-based use [48]. In practice, peroxygen products are effective when visible organic material is removed and contact times are met, and organic load can otherwise blunt disinfectant performance [49]. By contrast, organic acids act primarily by lowering pH, and their activity is highly pH-dependent, which limits reliability in paddocks with variable organic load [50]. Field evidence does not support soil alkalinization with lime as a control strategy for *R. equi* pneumonia; a multi-farm study found no association between surface soil geochemistry and disease risk [51]. These observations in our study are still exploratory because product, concentration, frequency, contact time, and soil conditions were not standardized. A sensible next step would be a prospective study that records the product and dose, measures soil pH before and after application, and tracks pre- and post-treatment viable counts based on *vapA* status.

To further contextualize the observed farm-level heterogeneity and to explore whether patterns were more compatible with clonal expansion or repeated introductions, genetic relatedness among *vapA*-positive isolates was assessed with PFGE. Substantial diversity was observed, with multiple clusters and numerous distinct pulsotypes, which is more consistent with the concurrent circulation of multiple genotypes than with domination by a single clone [52,53]. At the farm level, both clustered and unique profiles were detected, and in several foals the paired nasal and fecal isolates fell into different PFGE clusters, compatible with mixed-genotype colonization or repeated exposure. Conversely, instances in which a soil isolate clustered with a fecal isolate support the plausibility of environmental loading and shared exposure pathways [40]. In addition, some dam/foal pairs yielded closely related profiles, which is compatible with close-contact transmission and/or shared environmental sources; however, directional transmission and causality cannot be inferred from cross-sectional sampling.

When PFGE patterns were interpreted alongside the available epidemiological context, several exploratory signals emerged. Isolates from nearby premises tended to cluster in some instances, while other clusters included isolates from different provinces, a pattern compatible with both local environmental spread and longer-range connectivity through animal movements, shared personnel, equipment, or institutional networks [54]. These observations align with the broader concept that stud farm systems may experience repeated introductions superimposed on local amplification, but they remain hypothesis-generating given the cross-sectional design and incomplete movement/environmental covariates. Moreover, PFGE provides practical screening power but lower resolution than whole-genome sequencing; cluster assignment depends on band-matching parameters and similarity thresholds, and only *vapA*-positive isolates were genotyped [55]. Future longitudinal sampling combined with higher-resolution genomic typing and structured recording of animal movement and contact networks would help distinguish repeated introductions from on-farm persistence and would allow specific management measures to be evaluated against transmission-relevant endpoints.

Overall, susceptibility patterns were broadly favorable for agents commonly used in foal therapy. Non-susceptibility to macrolides and rifampin was uncommon in this dataset, whereas ampicillin non-susceptibility was frequent, and multidrug resistance was rare (2.7%). These findings are compatible with reports that resistance to first-line regimens remains variable across regions and farms and warrants continued culture-guided therapy and local surveillance, particularly in endemic settings where resistant lineages have been described [35,54]. Susceptibility was also compared between *vapA*-positive and *vapA*-negative isolates. No significant differences were detected for ampicillin, erythromycin, clarithromycin, or rifampin. These results suggest no clear signal of differential susceptibility based on *vapA* status in this panel, while recognizing that low numbers of non-susceptible isolates limit the power to detect modest differences. Interpretation of the disk diffusion results required methodological caution because CLSI disk diffusion breakpoints for *R. equi* are not available; accordingly, surrogate criteria were applied to support internal within-study comparisons, but direct benchmarking across studies using different methods or interpretive frameworks should be made cautiously.

This study was cross-sectional, so temporal dynamics, season-to-season persistence, and directionality of transmission could not be determined. Several potentially important farm- and environment-level covariates were not measured (e.g., herd/foal population size, soil characteristics, rainfall and wind patterns, and quantitative dust metrics), limiting the ability to evaluate drivers of between-farm variation. Province-level comparisons were also subject to confounding because farm participation and sampling intensity were necessarily uneven across locations. Finally, environmental sampling was not permitted on a subset of institutional farms, reducing completeness and comparability of soil and water results across all premises.

The dataset is strengthened by both its breadth and its integration, drawing on multiple farms across five provinces, paired nasal and fecal sampling from a large foal cohort, and environmental sampling where access was granted. Culture confirmation was complemented with virulence marker detection, PFGE-based genotyping of virulent isolates, and antimicrobial susceptibility testing. These laboratory findings were interpreted alongside structured, questionnaire-derived management information, enabling farm-level heterogeneity to be described in a way that is directly relevant to prevention and control on stud farms and providing a practical baseline for future longitudinal and genomic studies.

Strengths include multi-province enrollment, paired clinical and environmental sampling, integration of molecular and phenotypic testing, and a structured farm survey that situates results within a management context. Our data support basic biosecurity measures, including regular mechanical paddock cleaning, prompt isolation of clinically affected foals, and context-appropriate sanitation. Culture-guided therapy remains advisable; macrolide and rifampin should be reserved for clear indications, and ampicillin monotherapy should be avoided. Continued farm-level surveillance of overall positivity, *vapA* prevalence, and resistance is warranted. On stud farms in Türkiye, *R. equi* showed heterogeneous farm-level positivity, substantial genotypic diversity among *vapA*-positive isolates, and generally favorable susceptibility profiles for foal therapy. The management features align with the observed differences and should be evaluated prospectively. Future work should incorporate longitudinal sampling with standardized environmental metrics and genomic typing to distinguish repeated introductions from local expansion and to test specific hygiene measures.

## 5. Conclusions

This multi-province field study indicates that exposure to *Rhodococcus equi* is widespread on stud farms in Türkiye and varies substantially between premises. Approximately half of the confirmed isolates carried *vapA*, and PFGE revealed high genotypic diversity among virulent strains, supporting concurrent circulation of multiple pulsotypes rather than expansion of a single dominant clone. Susceptibility profiles were generally favorable for macrolides and rifampin, and multidrug resistance was rare; nevertheless, culture-guided therapy and continued antimicrobial stewardship remain essential when *R. equi* pneumonia is suspected. Taken together, these findings provide the first PFGE-based molecular baseline for virulent *R. equi* in Türkiye and support practical, farm-level prevention priorities, including regular mechanical paddock cleaning, prompt isolation of clinically affected foals, and context-appropriate sanitation. Future work should build on this baseline through longitudinal sampling with standardized environmental metrics and higher-resolution genomic typing to clarify transmission pathways, distinguish repeated introductions from local amplification, and evaluate targeted management interventions.

## Figures and Tables

**Table 1:** Positivity rates for *R. equi* in samples collected from horses and stud farms by province.

Samples	Provinces					*p* *χ* ^2^
	Malatya	Şanlıurfa	Bursa	İzmit	Eskişehir	
Soil	-	1/3 (33.3%)	2/2 (100.0%)	1/8 (12.5%)	1/4 (25.0%)	0.119 *χ*^2^ *=* 5.110
Water	-	1/3 (33.3%)	0/2 (0.0)	0/8 (0.0)	0/8 (0.0)	0.294 *χ*^2^ *=* 4.213
Fecal	8/25 (32.0%)^ab^	5/48 (10.4%)^c^	15/99 (15.2%)^bc^	42/113 (37.2%)^a^	28/143 (19.6%)^bc^	0.001 *χ*^2^ *=* 22.699
Nasal	4/25 (16.0%)^a^	7/48 (14.6%)^a^	2/99 (2.0%)^b^	21/113 (18.6%)^a^	9/143 (6.3%)^a^	0.001 *χ*^2^ *=* 21.466
Total	12/50 (24.0%)^ab^	14/102 (13.7%)^bc^	19/202 (9.4%)^c^	64/242 (26.4%)^a^	38/294 (12.9%)^c^	0.001 *χ*^2^ *=* 30.069

^a,b,c^: Values in the same row followed by different superscript letters differ significantly (*p* < 0.05). Group comparisons were performed using Pearson’s chi-square and Fisher’s exact tests.

**Table 2:** Distribution of *vapA* positivity among *R. equi* isolates by province.

Samples	Provinces					*p* *χ* ^2^
	Malatya	Şanlıurfa	Bursa	İzmit	Eskişehir	
Fecal	5/8 (62.5%)^ab^	1/5 (20.0%)^b^	5/15 (33.3%)^b^	12/42 (28.6%)^b^	24/28 (85.7%)^a^	0.001 *χ*^2^ *=* 26.581
Nasal	2/4 (50.0%)	4/7 (57.1%)	0/2 (0.0%)	6/21 (28.6%)	7/9 (77.8%)	0.068 *χ*^2^ *=* 7.895
Total positivity	7/12 (58.3%)^ab^	5/12 (41.7%)^b^	5/17 (%9.4%)^b^	18/63 (28.6%)^b^	31/37 (83.8%)^a^	0.001 *χ*^2^ *=* 31.566
Number of *vapA*-Positive Animals *	6/10 (60.0%)^ab^	5/11 (45.5%)^b^	5/17 (29.4%)^b^	17/52 (32.7%)^b^	28/32 (87.5%)^a^	0.001 *χ*^2^ *=* 27.604

^a,b^: Values in the same row followed by different superscript letters differ significantly (*p* <0.05). Group comparisons were performed using Pearson’s chi-square and Fisher’s exact tests. * Animals with *vapA*-positive *R. equi* detected in at least one nasal or fecal sample were considered positive.

**Table 3:** Summary phenotypes across 147 *R. equi* isolates.

No of Isolates	Isolates Susceptible to All Antibiotics Tested (%)	Isolates Resistant to Only One Antibiotic (%)	Resistant to Two Antibiotics (%)	Multiple (≥3) Resistant	Intermediate (%)
147	64 (43.5)	58 (39.4)	5 (3.4)	4 (2.72)	16 (10.8)

**Table 4:** Agent-specific disk diffusion interpretations for 147 *R. equi* isolates.

Antibiotics	Susceptible (%)	Intermediate (%)	Resistant (%)
Ampicillin (AMP)	73 (49.6)	7 (4.9)	67 (45.5)
Gentamicin (CN)	144 (98)	2 (1.3)	1 (0.7)
Vancomycin (VA)	146 (99.3)	1 (0.7)	0
Erythromycin (E)	141 (96)	2 (1.3)	4 (2.7)
Streptomycin (S)	132 (89.7)	4 (2.7)	11 (7.6)
Rifampin (RD)	137 (93.2)	5 (3.4)	5 (3.4)
Clarithromycin (CLR)	143 (97.3)	0	4 (2.7)
Tetracycline (TE)	146 (99.3)	0	1 (0.7)

**Table 5:** Farm management factors potentially relevant to *R.equi* epidemiology in participating stud farms (*N* = 17).

Variable	Category	Farms *n* (%)
Prior *R. equi* on farm	Yes	12 (70.6%)
	No	5 (29.4%)
Primary breed	English Thoroughbred	5 (29.4%)
	Arabian	4 (23.5%)
	Both	8 (47.1%)
Hyperimmune plasma in preventive protocol	Yes	2 (11.8%)
	No	15 (88.2%)
Accommodation	Own premises	11 (64.7%)
	Boarding	4 (23.5%)
	Both	2 (11.8%)
Isolation of clinically affected foals during treatment	Yes	14 (82.4%)
	No	3 (17.6%)
Hyperimmune plasma to all foals	Yes	1 (5.9%)
	No	16 (94.1%)
Other livestock kept on farm	Yes	8 (47.1%)
	No	9 (52.9%)
Bedding material	Straw	16 (94.1%)
	Shavings	1 (5.9%)
Sanitation agent (reported)	Virkon S	4 (23.5%)
	Lime powder	4 (23.5%)
	Organic acid	1 (5.9%)
	Grass medicine	2 (11.8%)
	Manure	2 (11.8%)
	None	4 (23.5%)
Foal deaths due to infection reported	Yes	10 (58.8%)
	No	7 (41.2%)
Bedding change (per week)	Once	2 (11.8)
	Three times	4 (23.5%)
	Every day	11 (64.7%)
Antibiotic treatment duration for foals (days)	Median (min–max)	15.0 (0–35)
Mechanical paddock cleaning (per year)	Median (min–max)	12.0 (0–24)
Disinfection (per year)	Median (min–max)	1.0 (0–5)
Dams of deceased foals previously produced infected offspring	Most	2 (11.8%)
	Rarely	3 (17.6%)
	None	8 (47.1%)
	Unknown	4 (23.5%)
Diet (categorized) *	Concentrate-only	1 (5.9%)
	Mixed	10 (58.8%)
	Forage-rich	6 (35.3%)

* Data are shown as n/N (%) unless otherwise indicated; percentages use *N* = 17 farms. Continuous variables are reported as median (min–max). Diet categories: Concentrate-only = commercial feed only; Mixed = concentrate + forage; Forage-rich = forage-heavy rations.

## Data Availability

The original contributions presented in this study are included in the article and Supplementary Material. Further inquiries can be directed to the corresponding author.

## Supplementary Materials

The following supporting information can be downloaded at https://www.mdpi.com/article/10.3390/vetsci13010072/s1: Table S1: Farm management questionnaire, response options, and coding schema. Items administered to veterinarians at the 20 participating stud farms; each question’s response options and the numerical coding used for analysis are listed; Table S2: Distribution of sampled animals by province and stud farm, categorized as healthy-appearing foals, clinically suspected foals, and dams (*N* = 428); Table S3: Concordance of *Rhodococcus equi* detection between clinically suspect foals and their dams based on paired nasal and/or fecal swabs (*n* = 36 dam-foal pairs); Table S4a: Farm-level management characteristics (Q1–Q9); Table S4b: Farm-level hygiene practices and outcomes (Q10–Q16); Table S5: Spearman rank correlation matrix (ρ) among questionnaire items (Q1–Q16) at the farm level. Entries show Spearman’s ρ for pairwise associations between items across farms (*n* = 17); Figure S1: Representative PFGE macrorestriction profiles of *vapA*-positive *Rhodococcus equi* after *AseI* digestion; Figure S2: Original image of Figure 2a; Figure S3: Original image of Figure 2b.
