# Supplementary material for: From Paddock to Foal: Prevalence and Genotypic Diversity of Rhodococcus equi on Stud Farms in Türkiye
Source: Vet Sci. 2026 Jan 10;13(1):72. doi: 10.3390/vetsci13010072 (PMC12846439; doi:10.3390/vetsci13010072)
Supplement: Supplementary file 1 [file vetsci-13-00072-s001.zip › vetsci-4032955-supplementary.pdf]

Table S1. Farm management questionnaire, response options, and coding schema. Items administered to veterinarians at the 20 participating stud farms; each question's response options and the numerical coding used for analysis are listed. Variables derived from this instrument are summarized in Table 5 and used in the correlation analyses in Table S5.

Item	Questionnaire item	Response options (suggested coding)
1	Has your farm experienced cases of *R. equi* infection in previous foaling seasons?	Yes/No (1/0)
2	Which breed(s) do you breed?	Thoroughbred, Arabian, Both (1/2/3)
3	Is hyperimmune plasma part of your routine preventive protocol?	Yes/No (1/0)
4	Are horses accommodated on your own premises or in boarding facilities?	Own premises/Boarding (1/2)
5	During treatment, are clinically affected foals isolated from other animals?	Yes/No (1/0)
6	Is hyperimmune plasma administered to all foals?	All foals/ Some foals / None (2/1/0)
7	Do you also rear species other than horses on the farm?	Yes/No (1/0)
8	What bedding material is used?	Wheat straw / Wood shavings / Other (specify) (1/2/3)
9	Which disinfectant/sanitizer is used for paddocks/stables?	Virkon® S / Organic acid / Lime powder / None / Other (specify) (1/2/3/0/4)
10	Have there been foal deaths attributed to *R. equi* on this farm?	Yes/No (1/0)
11	How often is bedding changed per week?	Numeric (times/week); or categories: Once / Twice / Three times / Every day (1/2/3/7)
12	What is the average duration of antimicrobial treatment for foals?	Numeric (days)
13	For dams of deceased foals, had previous offspring been affected?	Most / Some / Rarely / None / Unknown (4/3/2/1/9)
14	How many times per year is mechanical paddock cleaning performed?	Numeric (times/year)
15	How many times per year is disinfection performed?	Numeric (times/year)
16	What diet is provided to your horses?	Concentrate only/ Mixed (concentrate + forage) / Forage-rich (forage clearly predominant)

Table S2. Distribution of sampled animals by province and stud farm, categorized as healthy-appearing foals, clinically suspected foals, and dams (*N*=428).

Province / Farm	Healthy-appearing Foals	Clinically Suspected Foals	Dams	Total
**MALATYA**	21	2	2	25
A1	21	2	2	25
**ŞANLIURFA**	40	4	4	48
B1	21	2	2	25
C1	3	1	1	5
C2	16	1	1	18
**BURSA**	89	5	5	99
A3	36	2	2	40
B2	36	2	2	40
C3	17	1	1	19
**ESKİŞEHİR**	125	9	9	143
A2	42	7	7	56
B3	58	1	1	60
C4	5	-	-	5
C5	5	-	-	5
C6	15	1	1	17
**İZMİT**	81	16	16	113
B4	50	12	12	74
C7	2	-	-	2
C8	4	-	-	4
C9	4	3	3	10
C10	2	-	-	2
C11	14	1	1	16
C12	2	-	-	2
C13	3	-	-	3
**TOTAL**	**356**	**36**	**36**	**428**

Farm codes: A1-A3 and B1-B4, institutional stud farms; C1-C13, private stud farms; "-" indicates not sampled.

Table S3. Concordance of *Rhodococcus equi* detection between clinically suspect foals and their dams based on paired nasal and/or fecal swabs (n = 36 dam–foal pairs).

R. equi		Dam			*P*
		+	-	Total	χ^2
Foal	+	7	12	19	0.182
	-	3	14	17	χ^2=1.648
	Total	10	26	36	

Two-sided Fisher's exact test showed no significant association between foal and dam status (p = 0.182).

Table S4a. Farm-level management characteristics (Q1-Q9). Variables correspond to the questionnaire items in Table S1 (Q-codes shown in parentheses). Type denotes institutional (B) or private (C) farms.

Province	Farm code	Type	Q1 Prior *R. equi* history (Yes/No)	Q2 Breed (Thoroughbred/ Arabian/Both)	Q3 HIP in protocol (Yes/No)	Q4 Housing (Own/Boarding)	Q5 Isolate sick foals (Yes/No)	Q6 HIP to foals (All/Some/ None)	Q7 Other species (Yes/No)	Q8 Bedding (Straw/Shavings)	Q9 Disinfectant (Virkon S/Organic acid/Lime/None)
Şanlıurfa	B1	Institutional	Yes	English+Arabian	No	Boarding	Yes	None	No	Straw	Virkon S
Bursa	B2	Institutional	Yes	English+Arabian	No	Boarding	No	None	No	Straw	Virkon S
Eskişehir	B3	Institutional	Yes	English+Arabian	No	Boarding	Yes	None	No	Straw	Organic acid
İzmit	B4	Institutional	Yes	English+Arabian	Yes	Boarding	Yes	None	No	Straw	None
Şanlıurfa	C1	Private	No	English	No	Own	Yes	None	No	Straw	Lime powder
Şanlıurfa	C2	Private	Yes	Arabian	No	Own	No	None	Yes	Shavings	None
Bursa	C3	Private	No	English	No	Both	Yes	None	Yes	Straw	Other (manure)
Eskişehir	C4	Private	Yes	Arabian	No	Own	Yes	None	No	Straw	Lime powder
Eskişehir	C5	Private	No	English+Arabian	Yes	Own	Yes	All	No	Straw	Virkon S
Eskişehir	C6	Private	Yes	English+Arabian	No	Own	No	None	Yes	Straw	None
İzmit	C7	Private	Yes	English	No	Own	Yes	None	Yes	Straw	Other (Grass medicine)
İzmit	C8	Private	Yes	Arabian	No	Own	Yes	None	Yes	Straw	Manure
İzmit	C9	Private	Yes	English+Arabian	No	Own	Yes	None	No	Straw	Other (Grass medicine)
İzmit	C10	Private	No	English	No	Own	Yes	None	Yes	Straw	None
İzmit	C11	Private	Yes	English	No	Both	Yes	Some	Yes	Straw	Lime powder
İzmit	C12	Private	Yes	English+Arabian	No	Own	Yes	None	Yes	Straw	Lime powder
İzmit	C13	Private	No	Arabian	No	Own	Yes	None	No	Straw	Virkon S

Table S4b. Farm-level hygiene practices and outcomes (Q10-Q16).

Province	Farmcode	Type	Q10 Foal deaths due to *R. equi* (Yes/No)	Q11 Bedding change (/week)	Q12 Antimicrobial treatment duration (days)	Q13 Dam previously produced affected offspring (Most/Some/Rarely/None/Unknown)	Q14 Mechanical paddock cleaning (/year)	Q15 Disinfection (/year)	Q16 Diet (Concentrate/Mixed/Forage-rich/Other)	Animals sampled(n)	*R. equi*+, n/N (%)	*vapA* among *R. equi*+, n/N (%)
Şanlıurfa	B1	Institutional	Yes	7	10	Unknown	12	5	Concentrate	25	9/25 (36)	3/9 (33.3)
Bursa	B2	Institutional	Yes	7	35	Rarely	24	1	Forage-rich	40	8/40 (20)	1/8 (12.5)
Eskişehir	B3	Institutional	Yes	7	10	Unknown	12	1	Forage-rich	60	9/60 (15)	7/9 (77.7)
İzmit	B4	Institutional	Yes	7	21	Most	12	0	Forage-rich	74	27/74 (36.4)	8/27 (29.6)
Şanlıurfa	C1	Private	No	7	NA	None	12	1	Concentrate	5	0/5 (0)	0
Şanlıurfa	C2	Private	Yes	1	10	Unknown	0	0	Forage-rich	18	2/18 (11)	2/2 (100)
Bursa	C3	Private	No	3	7	None	24	1	Concentrate	19	1/19 (5.26)	0
Eskişehir	C4	Private	No	3	20	None	12	1	Concentrate	5	3/5 (60)	2/3 (66.6)
Eskişehir	C5	Private	No	7	NA	None	24	1	Mixed	5	0/5 (0)	0
Eskişehir	C6	Private	Yes	1	15	Most	2	0	Concentrate	17	6/17 (35.2)	6/6 (100)
İzmit	C7	Private	Yes	3	21	None	24	1	Concentrate	2	1/2 (50)	1/1 (100)
İzmit	C8	Private	No	7	10	Rarely	24	1	Forage-rich	4	4/4 (100)	2/4 (50)
İzmit	C9	Private	Yes	7	21	Rarely	12	1	Concentrate	10	4/10 (40)	0
İzmit	C10	Private	No	3	NA	None	12	0	Mixed	2	1/2 (50)	0
İzmit	C11	Private	Yes	7	15	Unknown	3	0.5	Concentrate	16	10/16 (62.5)	4/10 (40)
İzmit	C12	Private	Yes	7	21	None	3	0.5	Concentrate	2	2/2 (100)	2/2 (100)
İzmit	C13	Private	No	7	NA	None	24	1	Mixed	2	2/2 (100)	0

Variables correspond to the questionnaire items in Table S1 (Q-codes shown in parentheses). "*R. equi*+" denotes animals positive in ≥1 of nasal or fecal swabs; "*vapA* among *R. equi*+" is the percentage of *R. equi*-positive animals carrying *vapA*-positive isolates.

Table S5. Spearman rank correlation matrix (ρ) among questionnaire items (Q1-Q16) at the farm level. Entries show Spearman's ρ for pairwise associations between items across farms ($n = 17$). Only the upper triangle is displayed; the diagonal is omitted. Asterisks denote two-sided significance ($p < 0.05$; $p < 0.01$). Item wording and coding are provided in Table S1. Results are exploratory, and p-values are unadjusted for multiple comparisons. Notes: ρ, Spearman correlation coefficient; *p*, two-sided test.

	Q2	Q3	Q4	Q5	Q6	Q7	Q11	Q14	Q15	Q10	Q13
**Q1**	-0.426	-0.165	0.358	-0.299	-0.387	0.091	0.000	0.406	0.020	0.772**	0.070
**Q2**		-0.362	-0.665**	0.255	-0.248	0.467	0.358	-0.137	0.069	-0.473	-0.294
**Q3**			0.242	0.169	0.685**	-0.344	-0.264	-0.178	0.219	-0.065	0.278
**Q4**				-0.167	-0.060	-0.597*	-0.341	-0.058	-0.121	0.389	0.129
**Q5**					0.116	-0.182	-0.446	-0.318	-0.442	-0.387	-0.202
**Q6**						-0.236	-0.181	-0.298	-0.112	-0.299	-0.027
**Q7**							0.554*	0.268	0.456	0.070	-0.064
**Q11**								0.277	0.546*	-0.043	-0.014
**Q14**									0.508	0.492*	-0.233
**Q15**										0.131	0.344
**Q10**											-0.052

Significant associations. Q1 (prior farm history of *R. equi*) was strongly and positively correlated with Q10 (foal deaths attributed to *R. equi*) (ρ = 0.772, $p < 0.01$). In addition, Q14 (frequency of mechanical paddock cleaning) showed a positive correlation with Q10 (ρ = 0.492, $p < 0.05$). Item wording and coding for Q10 are given in Table S1; results are exploratory and *p*-values are unadjusted for multiple comparison.

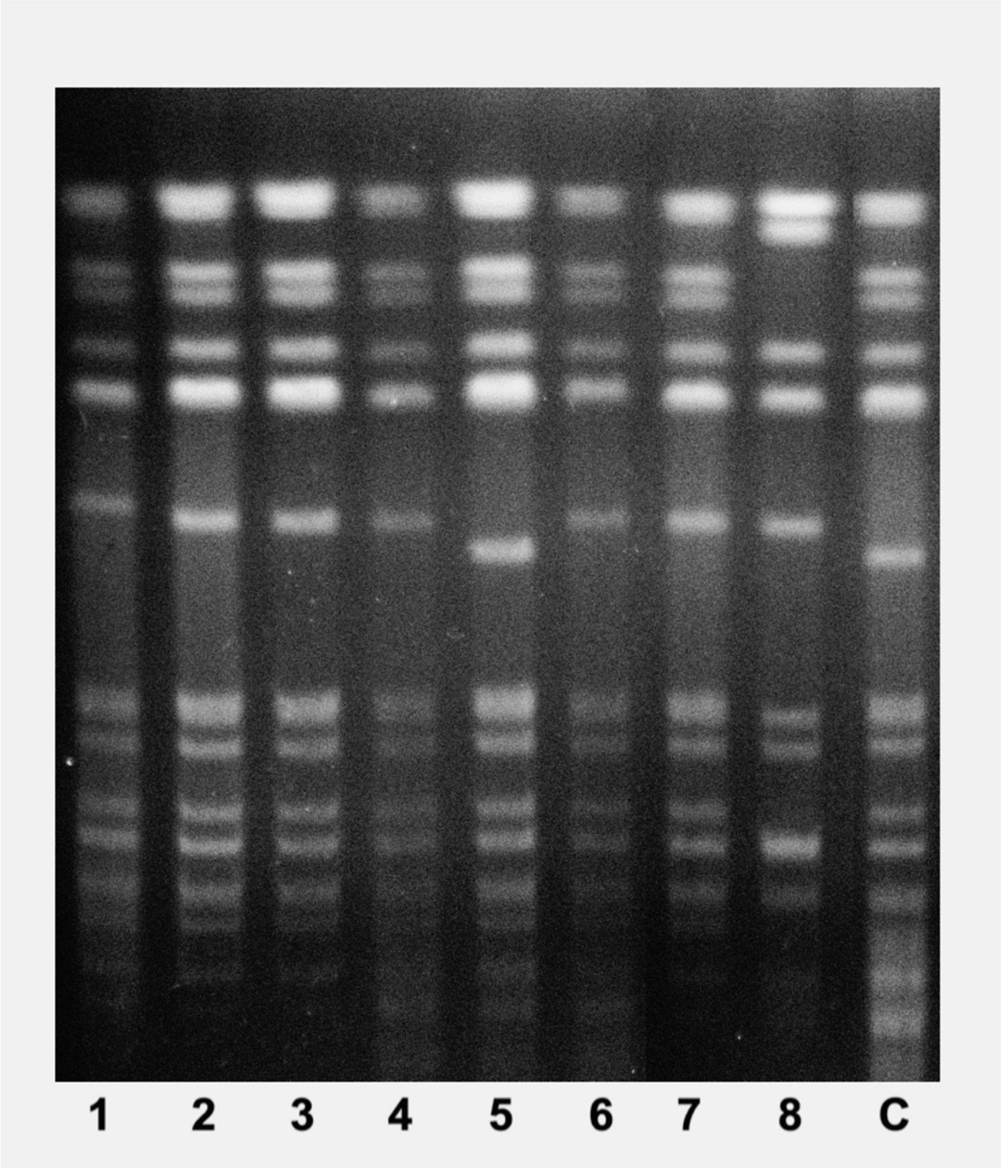

Figure S1. Representative PFGE macrorestriction profiles of *vapA*-positive *Rhodococcus equi* after *AseI* digestion. Lanes 1-8: field isolates; lane C: *R. equi* ATCC 33701 (process control; excluded from clustering). Lanes from different gels were combined for presentation, and inter-gel comparisons were interpreted cautiously. Similarities were calculated in GelCompar II using the Dice coefficient and UPGMA.

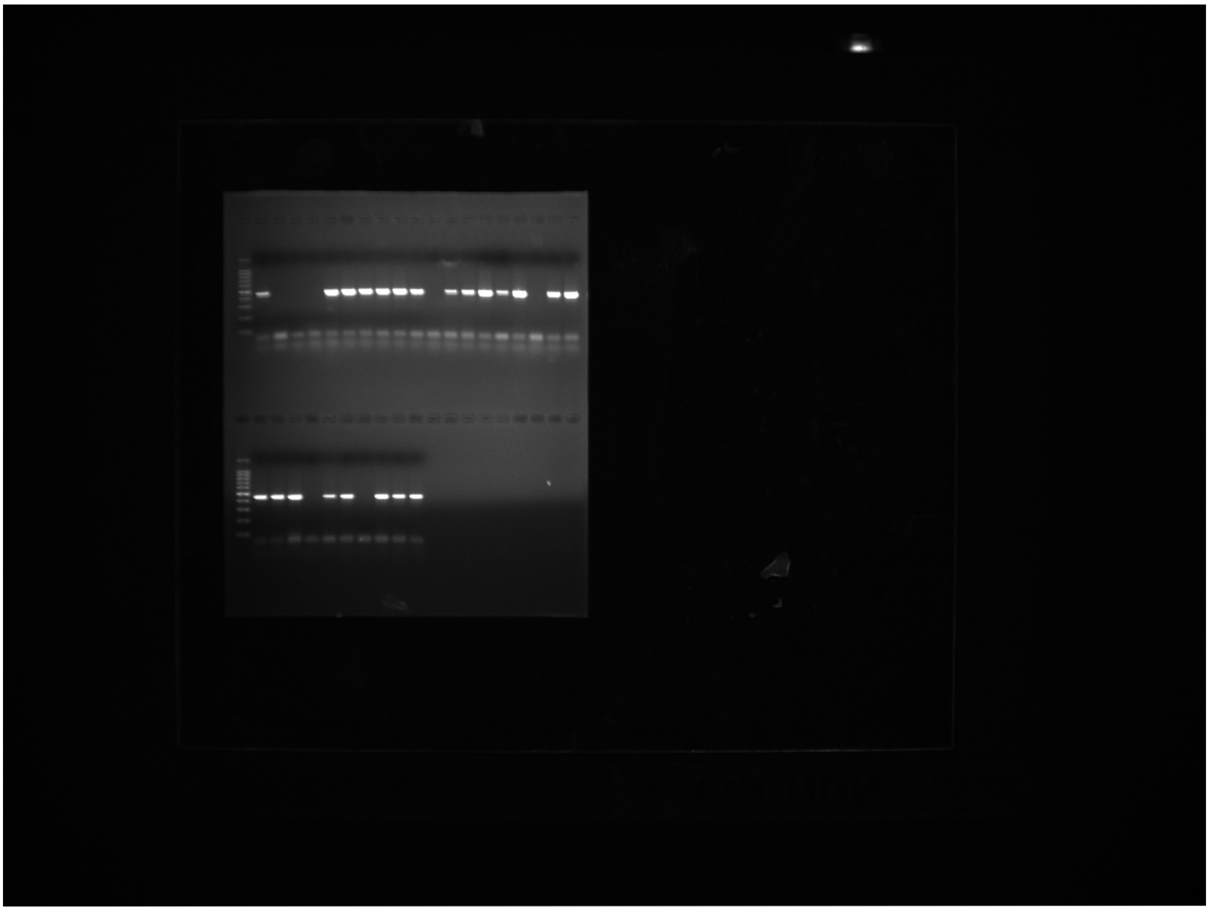

Figure S2. Original image of Figure 2a.

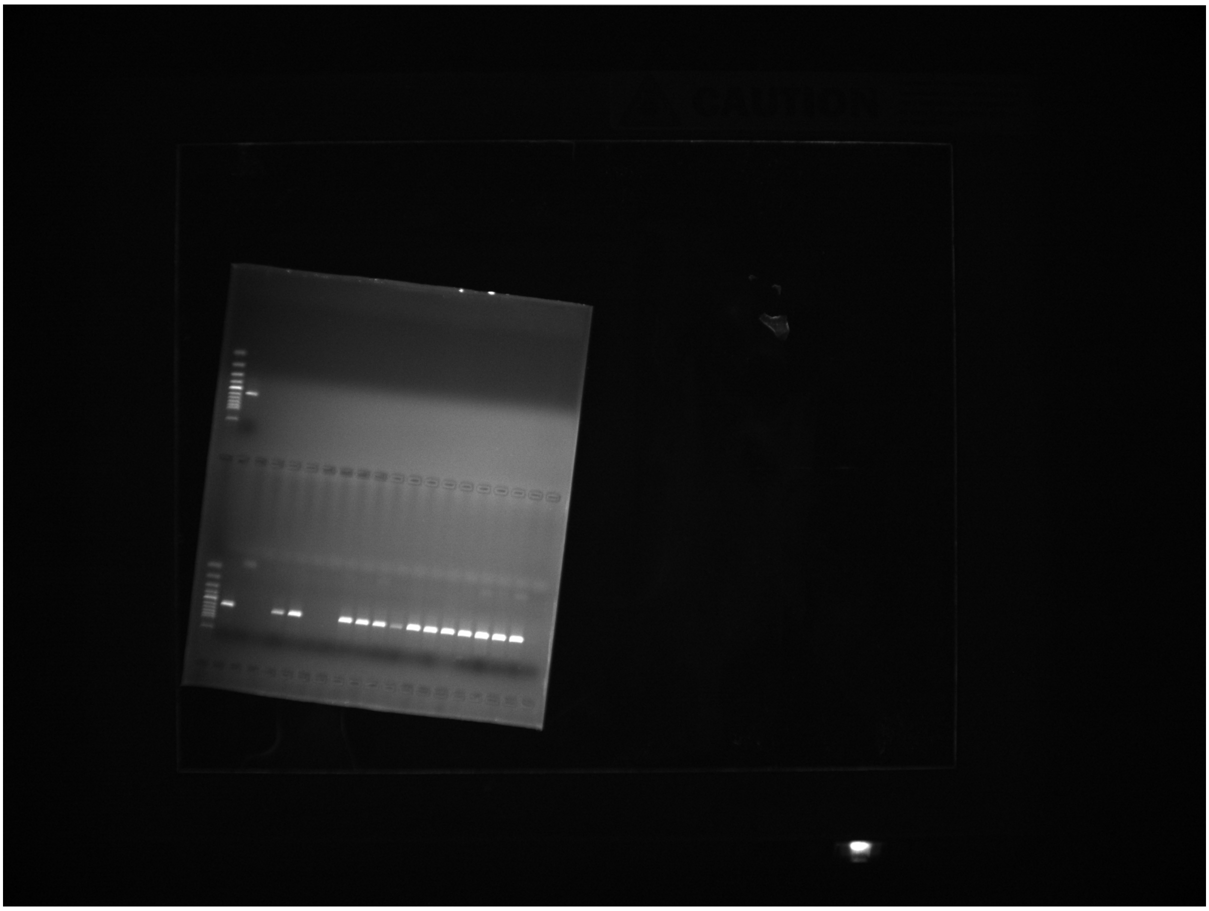

Figure S3. Original image of Figure 2b.
